# Causes and consequences of RNA:protein cross-links – lessons from chemotherapy

**DOI:** 10.1042/ETLS20253014

**Published:** 2025-12-08

**Authors:** Zornitsa Vasileva Kotopanova, Eloise Wilkinson, Zijian Zhang, John R.P Knight

**Affiliations:** 1 Division of Cancer Sciences, Faculty of Biology, Medicine and Health, The University of Manchester, Manchester, M13 9NT, U.K

**Keywords:** chemotherapy, reactive oxygen species, ribonucleoproteins, RNA, RNA-binding proteins, translation

## Abstract

Specific RNAs and proteins function together to impart function within cells. This ranges from binary interactions through to large macromolecular machines such as the ribosome. The emergence of the epitranscriptome over the past 20 years provides an excellent example of this relationship. The epitranscriptome is written on RNA molecules by protein-based enzymes, with these modifications required to diversify RNA function. The need for these functions in turn necessitates tight proximity between specific RNA transcripts and proteins. Here we describe an unwanted by-product of RNA:protein proximity – RNA:protein cross-links (RPCs). We describe how covalent bonds between RNAs and proteins form through one of two mechanisms. Throughout, we provide examples detailing clinical compounds that induce RPCs. The first mechanism of cross-linking is purely proximity-defined, occurring because of reactive third-party agents joining adjacent biomolecules. We discuss endogenous and exogenous agents that impart this activity and how the chemotherapeutic agent oxaliplatin induces cross-links by the same mechanism. The second class of RPCs forms between RNAs and specific RNA-modifying enzymes. These enzymes form transient covalent intermediates as part of their mechanisms of action, which we suggest can endure under certain conditions. We summarise evidence that the cancer drug 5-fluorouracil induces RPCs following its incorporation into RNA. Enzyme trapping occurs for specific modifier enzymes that target the non-canonical structure of the drug. Finally, we summarise recent work showing that cells contain specific molecular mechanisms to detect and resolve RPCs, placing this in the context of clinical cross-link induction.

## Introduction

RNA has many roles and localisations within cells and organisms. Messenger RNA (mRNA) acts as the genetic intermediate between the genome and proteome, while ribosomal RNA (rRNA) serves as the catalyst to enable this transfer of information. Structural, regulatory and cell-free RNAs expand the repertoire further. However, RNA seldom works alone, binding to specific proteins that are essential for its functions. These proteins range from transient regulatory RNA-binding proteins to longer lived RNA-protein interactions such as those in the ribosome. Additionally, as part of their relationship, specific RNAs and proteins form covalent bonds between each other. The best example of this being the RNA-modifying enzymes which almost ubiquitously form covalent intermediates with their substrates during the modification process. Like all enzymatic intermediates, these RNA:protein cross-links (RPCs) should be short lived.

In this mini review, we outline the causes and consequences of RPCs. This does not cover transient non-covalent interaction between RNAs and proteins. Although, as we discuss at the start of this review, this binding can give rise to proximity-defined cross-linking for several reasons. We also discuss enzymatic RPC events and detail evidence of clinical compounds that cause either enzymatic or proximity-defined cross-links. Finally, we outline the current understanding of how these cross-linking events are resolved.

### Proximity-defined RNA:protein cross-links

**Figure 1:**
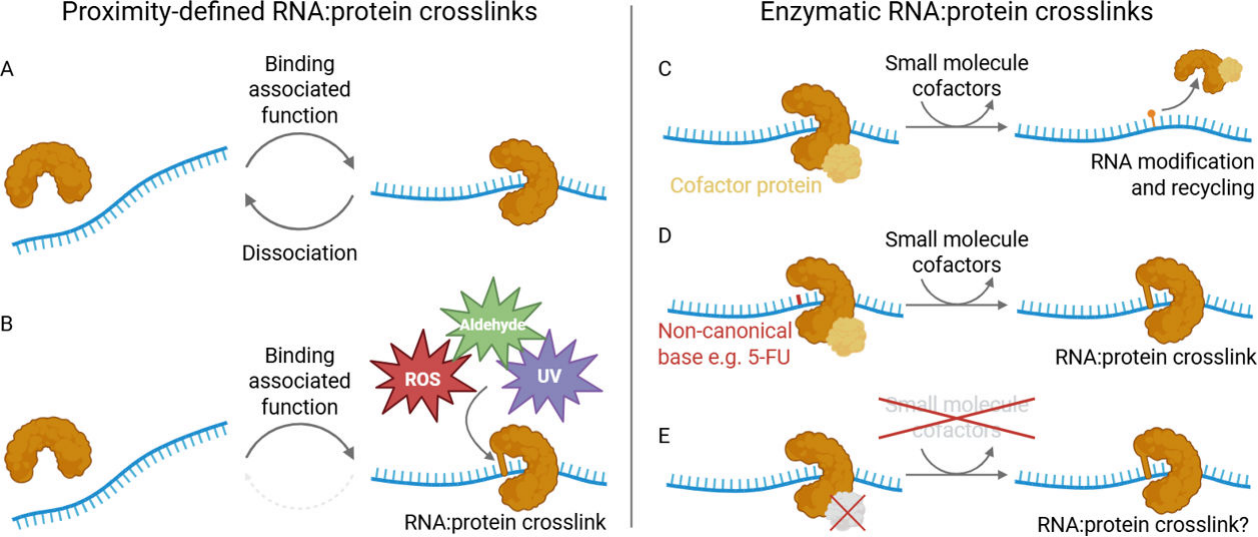
RNA:protein cross-links (RPCs) form by two mechanisms. (**A**) Proximity-defined cross-links form as a result of the functional association of RNAs and proteins as part of their function. These usually dissociate once function has been imparted. **B**) During normal association, RNAs and proteins can become covalently linked due to the action of third-party reactive factors – UV irradiation, reactive oxygen species (ROS) or aldehydes. **C**) Enzymatic cross-links form due to the catalytic activity of a protein upon an RNA substrate. This often requires protein and/or small molecule cofactors. Following catalysis, the enzyme dissociates from the now modified RNA. **D**) RNA-modifying enzymes can become covalently attached to RNA if they encounter a non-canonical nucleotide. Reactive nucleotides, such as 5-FU, promote this cross-linking. **E**) Cross-linking could occur when an enzyme attempts its catalytic function but then lacks certain cofactors to complete its function. The enzyme cannot progress catalysis beyond a covalent catalytic intermediate and is thus cross-linked. Figure created using BioRender. 5-FU, 5-fluorouracil.

RNA and protein are often found in close, non-covalent proximity. This close association can become covalent due to reactive third-party agents (Figure 1A–B). Below we outline the main three examples of these agents: aldehydes, reactive oxygen species (ROS) and ultraviolet (UV) radiation.

Aldehydes: The folate-driven one-carbon cycle releases formaldehyde, a potent cross-linking agent produced in large quantities within organisms [1]. Additionally, the human diet is a source of aldehydes. For example, acetaldehyde can be either directly ingested or produced as a consequence of the metabolism of alcohol in the liver [2]. The traditional focus of aldehyde cytotoxicity has centred on the induction of DNA damage. However, in 2023, Rahmanto et al. demonstrated that formaldehyde induces RPCs, which stalls ribosomes and inhibits translation [3].

ROS: ROS and free radicals play a crucial role in normal cellular homeostasis. However, due to their high reactivity, they can also rapidly interact with biomolecules such as nucleic acids, proteins and lipids, significantly impacting their biological functions. ROS-induced damage to RNA is multifaceted, including oxidative modifications such as 8-oxo-guanosine, cross-linking and strand breaks [4]. ROS have different affinities for the four nucleobases, depending on their structure. Guanosine is the most reactive base and as such is the dominant site of RNA damage [5].

In 2006, Regnier et al. observed in yeast that RPCs aggregate as a result of oxidative stress, with cytoplasmic ribosomal proteins (RPs) being the most susceptible to oxidation [6]. However, mitochondria are the main source of the ROS within our cells. Indeed, Tanaka et al. demonstrated that hydrogen peroxide induces cytochrome c to cross-link with oxidised RNA in a concentration-dependent manner [7]. This process facilitates the dissociation of cytochrome c from mitochondria into the cytosol, thereby initiating mitochondria-mediated apoptosis under oxidative stress conditions. It is interesting to note that the human mitochondrial transcriptome contains only 7% guanosine, implicating an evolutionary pressure to favour the lower reactivity of the other bases in this highly oxidative environment [8].

We recently found that RPCs are induced by a combination of ROS and the proximity afforded by liquid–liquid phase separation (LLPS) [9]. Using RNA- and protein-rich stress granules (SGs) as a model, we found more RPCs following SG induction during osmotic shock. Importantly, these RPCs were dependent on ROS, as when ROS levels were reduced with a scavenger compound, RPCs were not induced despite retention of LLPS. We proposed that the functional benefits of LLPS outweigh the detrimental effects of RPCs [9].

UV irradiation: UV has multiple effects on RNA, including photochemical base modifications, such as pyrimidine dimers [10], RNA–RNA cross-linking, oxidative damage and RPC formation [11]. It was first reported in the 1970s that UV induces RPCs in insect eggs [12]. In 2004, Casati et al. reported in maize that UV radiation cross-linked RNA with specific RPs, accompanied by a reduction in translation, and concluded that RNA was also the main target of UV radiation [13]. In fact, UV-induced RPCs and RNA-RNA cross-links are widely used by researchers in cross-linking and immunoprecipitation experiments to explore RNA–protein and RNA–RNA interactions [14]. Despite this, the physiological relevance of UV cross-linking is an important point to consider. The UV doses used in laboratory experiments exceed those that will be experienced by most, if not all, mammalian cells. The RPCs formed by UV in the plant kingdom are likely to be more representative of their physiological setting. Nevertheless, UV-induced RPCs remain a reliable tool and model system for the analysis of RPCs.

### Proximity-defined RPCs induced by oxaliplatin

Oxaliplatin and the two other approved platinum drugs, cisplatin and carboplatin, were all canonically considered DNA-damaging agents that produce platinum-DNA adducts [15]. These agents preferentially bind to the nitrogen atom at position seven of guanosine and more seldomly adenosine [16]. All three drugs are bivalent, meaning they can co-ordinate two positions on the biomolecule(s) with which they form adducts [17]. Chemically, the sugar ribose backbone of RNA is innately more reactive than the deoxyribose in DNA. Additionally, the nucleobases are identical except for the use of the more reactive uridine in RNA than the thymidine in DNA. Cells contain around 10-fold more RNA than DNA, and RNA is more widely distributed within, upon and outside cells. These points lead to the argument that damage inflicted on RNA is more likely and spatially pervasive than DNA damage [5]. Adding more complexity, a recent study illustrated that RNA damage can in fact lead to DNA double-strand breaks if not surveyed and repaired properly [18].

Recently, there has been a shift away from DNA damage as the sole mechanism of action (MOA) of oxaliplatin. It remains likely that oxaliplatin-induced damage of DNA contributes to the cytotoxicity, but it is now clear that this is not the only mechanism determining cell fate. Equimolar concentrations of the platinum agents lead to different amounts of DNA adducts, with oxaliplatin lesions being outnumbered by those caused by cisplatin [15,19,20]. Moreover, the three drugs are used to treat different tumour types with oxaliplatin originally effective against colorectal cancer [21,22] and now also used for pancreatic and oesophageal cancers. Whereas cisplatin and carboplatin have long been used for patients diagnosed with testicular, ovarian and lung malignancies [23]. Additionally, oxaliplatin can be effective against some cisplatin-resistant tumours and is associated with a distinct side effect profile [24,25]. Together, this indicates that oxaliplatin has different molecular targets to the other platinum agents, with this underlying its divergent clinical effects.

**Figure 2:**
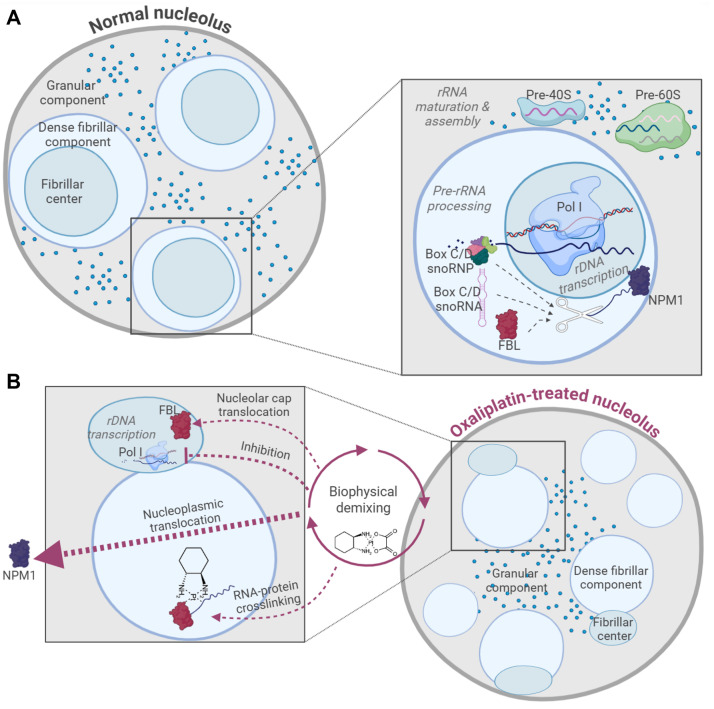
Nucleolar effects of oxaliplatin. **A.** The normal nucleolus is phase separated into fibrillar centre (FC), dense fibrillar component (DFC) and granular component (GC). rDNA transcription is conducted by polymerase I (Pol I) in the FC, whereas the DFC is the site of pre-rRNA processing, which can occur co-transcriptionally and can be brought about by box C/D small nucleolar ribonucleoproteins (snoRNPs) guided by box C/D small nucleolar RNAs (snoRNAs), positioning the methyltransferase fibrillarin (FBL) towards the target pre-rRNA, which is illustrated alongside nucleophosmin 1 (NPM1), acting as a molecular chaperone. Maturation of the rRNAs and their assembly into small pre-40S and large pre-60S ribosomal subunits occurs in the GC. **B.** By contrast, the oxaliplatin-treated nucleolus is characterised by loss of integrity, rDNA transcription inhibition, FBL translocation into nucleolar caps, nucleoplasmic translocation of NPM1 and RNA:protein cross-linking with the DFC as a suspected main site of action of the drug [29]. Figure created using BioRender.

Oxaliplatin is a class 2 RNA-damaging agent, meaning that its binding to RNA is driven by localisation and proximity [5]. rRNA makes up ~85% of the RNA by mass within cells [26], making this the most likely target for these compounds. rRNA functions within ribosomes in association with RPs in the cytoplasm. The same rRNA sequences are present at high concentrations within the nucleoli, the LLPS molecular factories that synthesise ribosomes. A hallmark study conducted by Rubbi and Milner highlighted the role of the nucleolus as a stress sensor, demonstrating a strong p53 response upon UV irradiation of the nucleolus but no p53 accumulation upon UV irradiation of the nucleoplasm [27]. In the context of chemotherapy, yeast depleted of nucleolar factors is more sensitive to chemotherapeutics [28]. Synthesis of rRNA is blocked by oxaliplatin at clinically relevant concentrations, reflecting the strong inhibitory effect of the drug on rRNA transcription, accompanied by loss of nucleolar integrity (Figure 2) [30]. Although cisplatin was reported to have similar effects, this only occurs above clinically relevant doses.

Consistent with these previous reports, a functional genetic approach revealed that oxaliplatin more closely resembles transcription-translation inhibitors, while cisplatin was confirmed as a DNA cross-linker [31]. Exemplifying this, comet assays show less DNA damage by oxaliplatin when compared with cisplatin or no-treatment control. However, oxaliplatin reduced protein synthesis within 9 h of treatment, an effect not seen with cisplatin. The counterintuitive reduction in DNA damage after oxaliplatin treatment was attributed to fewer cells being in S phase and therefore having fewer partially synthesised, mobile DNA fragments. This work confirmed the different MOAs between the platinum compounds that had previously been implied by their divergent clinical use. Furthermore, tumours commonly treated with oxaliplatin exhibited higher expression of genes involved in the ribosome pathway than tumours commonly treated with cisplatin [31]. This raises questions around chemotherapies being innately ‘non-targeted’, as if this were the case, they would work equally well in all tumour settings, and their efficacy would not correlate with expression of certain genes. Indeed, the obvious conclusion is that these genes signpost the targets of the drug (i.e., ribosome synthesis) with the name ‘multi-targeting therapy’ perhaps being more accurate.

The redistribution of nucleolar proteins in response to oxaliplatin, and not cisplatin or carboplatin, was also reported by [32]. Building on this, the same authors demonstrated the rapid inhibition of rRNA synthesis following oxaliplatin treatment, which coincides with the first relocalisation events of nucleolar proteins [33]. Again, cisplatin did not have the same effect but resulted in a more pronounced DNA damage response. Given the LLPS that occurs within nucleoli, Schmidt et al. asked how the different platinum agents altered the physical properties of the organelle [29]. They found that oxaliplatin, but not cisplatin, increased the surface tension of nucleoli and resulted in nucleolar demixing. An increase in the nucleolar optical thickness was also observed in cancer cells following oxaliplatin treatment to a greater extent than cisplatin treatment using digital holographic microscopy [9]. Together, these results illustrate the profound physical changes that occur within nucleoli following oxaliplatin treatment. But what molecular factors underlie these changes? Of note, these nucleolar changes were recently shown to be less reversible than nucleolar changes caused by other nucleolar stress inducers such as actinomycin D [34]. This suggests that oxaliplatin produces harder to resolve nucleolar changes, suggestive of covalent damage.

The molecular effects of platinum agents on nucleolar nucleic acids and proteins were tested by [29]. Oxaliplatin formed adducts with recombinant fibrillarin (FBL) or nucleophosmin 1 (NPM1), more so than cisplatin. Furthermore, mixing FBL and NPM1 with a population of single- and double-stranded DNA and single-stranded RNA revealed preferential affinity towards FBL over nucleic acids. Thus, the ability of oxaliplatin to damage nucleolar scaffold components was argued to determine the demixing of nucleoli. We recently extended this observation to show that oxaliplatin can utilise its bivalency to induce RPCs, including specific protein factors [9]. Aqueous identification of RNA elements was used to detect RPCs involving nucleolar proteins such as FBL, dyskerin (DKC) and RPS6. Liquid chromatography-mass spectrometry analysis of RPC proteins showed enrichment of 90S preribosome factors following oxaliplatin treatment. This was validated by showing pre-rRNA sequences found in the 90S preribosome are more susceptible to adduct formation than other abundant RNAs using a reverse-transcription-based stalling assay. Pre-rRNAs are innately rich in guanosines, the preferred target of oxaliplatin. This led to a hypothesis that the damage detected within pre-rRNAs was simply a product of their high guanosine content.

### Enzymatic RNA:protein cross-links

The 5-fluorouracil (5-FU) is an antimetabolite chemotherapeutic agent used to treat over 2 million cancer patients each year [35,36]. The drug was first synthesised and published by Heidelberger et al. in 1957, following the discovery of increased uracil uptake in hepatic carcinoma cells in rats [37,38]. The drug forms the backbone of modern combination therapy for many carcinomas and adenocarcinomas due to its efficacy and affordability. 5-FU is a uracil analogue in which the normal carbon-5 hydrogen atom is substituted with a fluorine atom. It is this hydrogen that is frequently modified by RNA writer enzymes, which change RNA function [39].

5-FU and its metabolites are mistaken for uracil or thymine and are thus incorporated into both RNA and DNA [40]. The main cytotoxic mechanism of 5-FU has long been hypothesised to act through the cross-linking and subsequent inhibition of thymidylate synthase (TS) by the 5-FU metabolite fluorodeoxyuridine monophosphate [35,41,42]. This inhibition of TS prevents the formation of 2′-deoxythymidine-5′-monophosphate, leading to nucleotide pool imbalance and DNA damage. A recent study found that 5-FU resistant cells depend on high levels of TS for survival, with the conclusion that the TS protein sequesters 5-FU metabolites away from other mechanisms such as RNA incorporation [43].

5-FU is a class 1 RNA-damaging agent, meaning that it, or one of its metabolites, is incorporated into RNA by RNA polymerases [5]. The incorporation of 5-FU into RNA has been directly correlated with drug response across a panel of sensitive and resistant human cell lines [44]. This study revealed inhibition of 5-FU incorporation into RNA significantly reduced the efficacy of the drug, strongly emphasising an RNA-mediated mechanism of cytotoxicity for 5-FU. It is therefore interesting to note that cross-linking mechanisms, analogous to the cross-link inhibition of TS that occurs upon 5-FU treatment, also occur following incorporation into RNA [9,45]. In fact, the ability of 5-FU to form aberrant cross-links between RNA-modifying enzymes, such as transfer RNA (tRNA) methyltransferase 2 homolog A (TRMT2A) and pseudouridine synthase 1 (PUS1) is well established across multiple organisms [46,47] (Figure 1C–D). Separate research on *Saccharomyces cerevisiae* also revealed strains with haplo-insufficiencies in tRNA-modifying enzymes such as Trmt2a, dihydrouridine synthase 3 (Dus3) and Pus1 displayed significantly increased sensitivity to 5-FU treatment [48].

In mammals, 5-FU has been found to cross-link different RNA writer enzymes to their RNA substrates, many of which are homologous to cross-linked enzymes found in other species. Specific examples of these enzymes are DKC (pseudouridine writer), TRMT2A (5-methyluridine writer), and dihydrouridine synthase like (DUS3L dihydrouridine writer) that have been found to cross-link to fluorinated RNA in HCT116 cells at clinically relevant dosages of 5-FU [9,48–50]. The commonality between all these writer enzymes is that each one targets their modifications onto the fifth carbon of the uracil ring, which in 5-FU has a fluorine in place of a hydrogen. Therefore, when targeted by these writer enzymes results in irreversible covalent bond formation. Taken together, these results emphasise a novel, RNA-based mechanism by which 5-FU may drive cytotoxicity through writer enzymes getting ‘stuck’ whilst trying to catalyse their canonical modifications.

**Figure 3:**
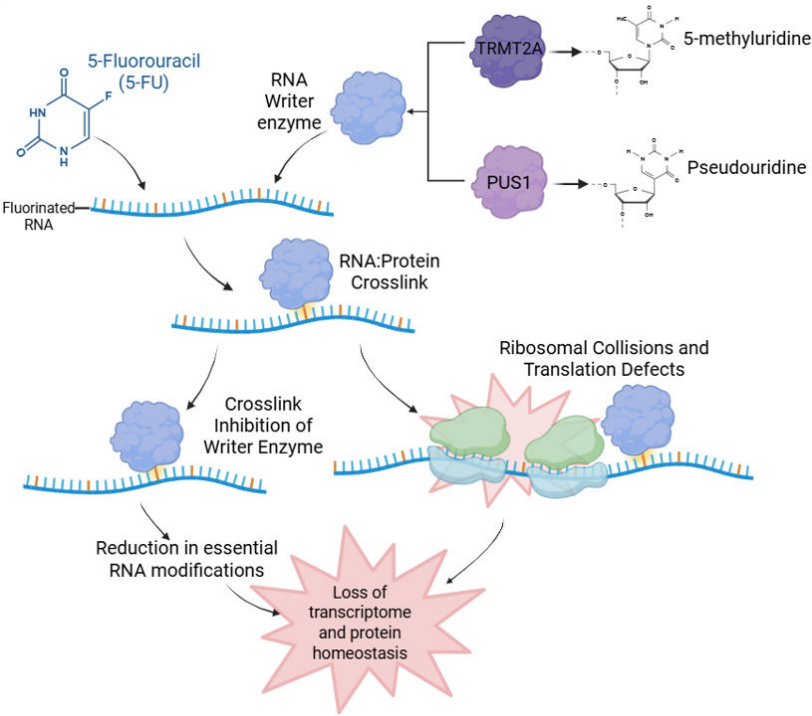
RNA:protein cross-links (RPCs) induced by 5-fluorouracil (5-FU). 5-FU-incorporated RNA forms RPCs with writer enzymes such as transfer RNA (tRNA) methyltransferase 2 homolog A (TRMT2A) and pseudouridine synthase 1 (PUS1). This has two effects – the cross-link inhibition of these writer enzymes and the induction of ribosomal collisions. Ultimately, these RPCs result in a reduction in essential post-transcriptional RNA modifications such as pseudouridine and 5-methyluridine and overall loss of proteostasis. Figure created using BioRender.

The downstream effects of these RPCs are characterised for some RNA and protein pairs, yet we do not know the exact contribution of protein loss of function compared with RPC gain of function. Research has displayed that 5-FU treatment results in significantly reduced levels of the RNA modifications produced by these writer enzymes [51], indicating a loss of protein function. Following treatment with 5-FU, significantly less m5U and pseudouridine (Ψ) modifications were observed in both tRNA and rRNA [51]. These modifications, as mentioned above, are catalysed by TRMT2A and pseudouridine synthases such as DKC and PUS1, which are known to cross-link to RNA containing 5-FU (Figure 3) [9,45,51]. This cross-link inhibition produced by RPCs results in widespread cellular dysfunction, such as impaired ribosome biogenesis and mRNA processing, and more generally impaired proteostasis [52–54].

Together, this provides evidence for a novel MOA of 5-FU, mediated by its incorporation into RNA, resulting in the formation of RPCs. These cross-links form at naturally occurring protein–RNA interaction interfaces and result in downstream disruption of essential protein and RNA function, potentially contributing to cell death. It is currently unclear if it is the loss of protein function, the loss of RNA function or the gain of aberrant RPCs that contributes most to cytotoxicity. Exactly the same questions can be posed for oxaliplatin, which is commonly used in clinical combination with 5-FU.

Here, we use the RPCs induced by 5-FU to demonstrate their enzymatic induction. However, enzymatic RPCs can also form in the absence of drug treatment. Indeed, Zhang et al. reported significant RPCs in the absence of drug treatment. An unbiased identification of the cross-linked RNA proteome identified more than 2000 proteins [9]. Of these, 21 were RNA-modifying enzymes and 19 were aminoacyl-tRNA synthetases, which are two example classes of proteins that form covalent intermediates with RNA during their mechanisms of action. This prompts questions about factors that may influence endogenous enzymatic RPC abundance (Figure 1E). Many RNA-modifying enzymes use cofactors, so how does their availability affect RPCs? Similarly, RNA-modifying enzymes often form multimeric protein complexes, so what happens if one subunit is absent or dissociates? The data also imply that proximity RPCs are more prevalent than enzymatic RPCs. Further work is needed to understand the RPC proteome and its prevalence in other model systems.

### RNA:protein cross-link resolution

**Figure 4:**
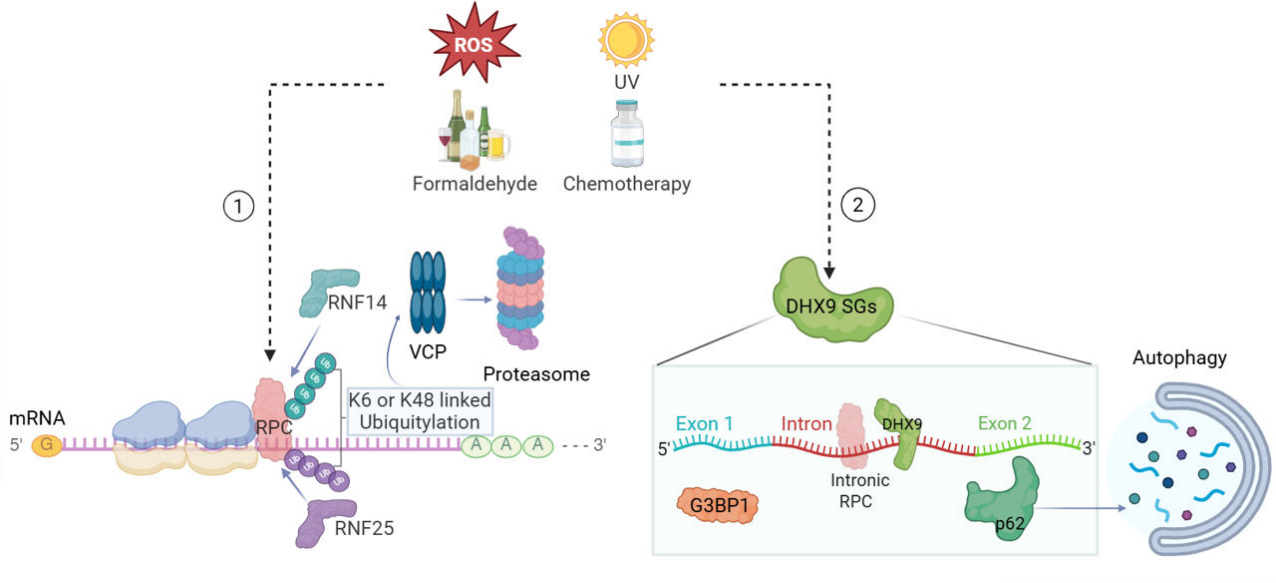
Two pathways of RNA:protein cross-link (RPC) resolution. RPC resolution via ubiquitin-mediated degradation begins when translating ribosomes encounter RPCs, triggering the recruitment of several key proteins such as ring finger protein 25 (RNF25) and ring finger protein 14 (RNF14). RNF14 subsequently catalyses K6- and K48-linked ubiquitylation of RPCs, marking them for degradation by the valosin-containing protein (VCP)-dependent proteasomal pathway. 2. Stressors such as ultraviolet (UV) irradiation can induce the formation of DExH-box helicase 9 (DHX9)-positive stress granules (SGs) in daughter cells, which are enriched with intronic RPCs. The autophagy receptor p62 is activated and facilitates the disassembly of these DHX9 SGs. Figure created using BioRender.

Studies of RPC resolution have primarily used aldehydes, ROS or UV as tools for their induction. These have wide-reaching effects on many biomolecules, leading Rahmanto et al. and Zhao et al. to use photoactivated ribonucleoside enhanced cross-linking to specifically induce RPCs [3,55]. These studies found that RPCs in mRNAs are specifically recognised by translating ribosomes, which are then marked by atypical K6-linked ubiquitylation. This process is catalysed by the E3 ubiquitin ligase ring finger protein 14 with the ultimate outcome being the RPC being degraded by valosin-containing protein and the proteasome [3,55] (Figure 4). The same studies found that neither oxidative stress nor the alkylating agent methyl methanesulfonate induces K6 ubiquitination ligation, indicating that this is a specific response to RPCs, distinct from other RNA damage.

Recently, Zhou et al. reported that UV induces RNA, but not DNA, cross-linking damage, triggering the assembly of DExH-box helicase 9 (DHX9) SGs enriched in proteins cross-linked to intronic RNA [56]. Intron cross-linking damage prevents pre-mRNA-binding proteins from dissociating from pre-mRNA, thereby blocking spliceosome access and impairing RNA splicing and processing. In daughter cells, DHX9 SGs play a protective role against parental RNA damage. Intronic RPCs activate a double-stranded RNA (dsRNA) response in daughter cells, with DHX9 regulating the abundance of dsRNA within DHX9 SGs. This shields these cells from the harmful innate immune response to dsRNA, but not the shutdown of translation mediated by protein kinase R. This is thought to allow cells to resolve RPCs. Resolution appears to use autophagy given the enrichment of the autophagy receptor p62 in DHX9 SGs (Figure 4).

Recent studies have also investigated the RNA responses to 5-FU treatment. To interpret these studies, we must remember that as well as inducing RPCs, 5-FU incorporation into mRNA also disrupts translation, causing ribosome collisions [57]. Whether these collisions occur on 5-FU in mRNA or RPCs on mRNA remains to be determined. Whatever the cause, collisions activate the ribotoxic stress response (RSR) and the integrated stress response (ISR), as well as ribosome-associated quality control (RQC), which aim to resolve these collisions and deal with the nascent peptides [58,59]. Downstream responses to ISR and RSR activation are reduced global mRNA translation and cell cycle arrest; persistent activation of these pathways can also lead to apoptosis [58,60,61]. The E3 ubiquitin ligase zinc finger protein 598 (ZNF598), which plays an important role in RQC by monoubiquitinating RPs[62–64], has been implicated in the cytotoxicity of 5-FU treatment [57]. These findings emphasise the role of downstream degradative machinery in 5-FU resistance.

Notably, the autophagosome has also been linked to poorer drug responses, with its inhibition improving drug response [65,66]. More specifically, lysosomal-dependent autophagy is an important process through which cells deal with damaged rRNA following 5-FU treatment [44]. rRNA can be degraded by endo- and exo-nucleases, raising the question of why the autophagy pathway is activated to degrade 5-FU:RNA. Could this be a result of RPCs, which would not be suitable substrates for nucleases? Chen et al. found that inhibiting autophagy increased cell viability following 5-FU metabolite treatment. The reasons for this require further investigation in the context of RPCs.

This progress in RPC resolution has to date focused on (pre-)mRNA cross-links or not precisely reported the RNA or protein parties. Future work should reveal how the current pathways, or yet unknown pathways, are engaged to manage specific cross-links. We anticipate future work identifying the determinants of which resolution pathway(s) are utilised. Is this decided by the type of RNA cross-linked, or the exact protein or both parties? Or is the source of the cross-link important, such as the proximity and enzymatic cross-links outlined here?

**Summary points**

- RNA:protein cross-links (RPCs) can form because of proximity and damaging reactive agents or due to endogenous enzymatic activity.
- Both proximity-defined and enzymatic RPCs are promoted by oxaliplatin and 5-FU respectively.
- Cells contain mechanisms to sense, respond to and resolve RPCs to mitigate their impact.
- Understanding what determines endogenous and (drug-)induced RPC formation could reveal novel disease-targeting mechanisms for exploitation.

## Open Access

This article has been published open access under our Subscribe to Open programme, made possible through the support of our subscribing institutions, learn more here: https://portlandpress.com/pages/open_access_options_and_prices#conditional

## Abbreviations

DHX9: DExH-box helicase 9
DKC: dyskerin
Dus3: dihydrouridine synthase
FBL: fibrillarin
FC: fibrillar centre
5-FU: 5-fluorouracil
ISR: integrated stress response
LLPS: liquid–liquid phase separation
MOA: mechanism of action
NPM1: nucleophosmin 1
PUS1: pseudouridine synthase 1
ROS: reactive oxygen species
RPCs: RNA:protein cross-links
RPs: ribosomal proteins
RQC: ribosome-associated quality control
RSR: ribotoxic stress response
SGs: stress granules
TRMT2A: tRNA methyltransferase 2 homolog A
TS: thymidylate synthase
UV: ultraviolet
dsRNA: double-stranded RNA
mRNA: messenger RNA
m5U: 5-methyluridine
rRNA: ribosomal RNA
tRNA: transfer RNA
